# Coherence and resonance effects in the ultra-intense laser-induced ultrafast response of complex atoms

**DOI:** 10.1038/srep18529

**Published:** 2016-01-06

**Authors:** Yongqiang Li, Cheng Gao, Wenpu Dong, Jiaolong Zeng, Zengxiu Zhao, Jianmin Yuan

**Affiliations:** 1 Department of Physics, College of Science, National University of Defense Technology, Changsha 410073, Hunan, P. R. China; 2 IFSA Collaborative Innovation Center, Shanghai Jiao Tong University, Shanghai 200240, P. R. China

## Abstract

Both coherent pumping and energy relaxation play important roles in understanding physical processes of ultra-intense coherent light-matter interactions. Here, using a large-scale quantum master equation approach, we describe dynamical processes of practical open quantum systems driven by both coherent and stochastic interactions. As examples, two typical cases of light-matter interactions are studied. First, we investigate coherent dynamics of inner-shell electrons of a neon gas irradiated by a high-intensity X-ray laser along with vast number of decaying channels. In these single-photon dominated processes, we find that, due to coherence-induced Rabi oscillations and power broadening effects, the photon absorptions of a neon gas can be suppressed resulting in differences in ionization processes and final ion-stage distributions. Second, we take helium as an example of multiphoton and multichannel interference dominated electron dynamics, by investigating the transient absorption of an isolated attosecond pulse in the presence of a femtosecond infrared laser pulse.

Master equation approach is a standard technique for open quantum systems^1^ and a successful theory in descriptions of light-matter interactions, such as in condensed matter physics^2^, chemistry and biology^3^, quantum optics^4^, and ultracold gases^5^. The master equation is quite general and encompasses various physical phenomena, as long as these phenomena share common physical mechanisms, i.e. the interplay between coherence and dissipation. Traditionally, these kinds of systems are treated within the framework of few-level models^5^,^6^,^7^. With the development of highly bright lasers^8^, energy can be deposited in a broad range in the laser induced systems, which normally relax in a vast number of decay channels. In this case, new challenges appear for dynamics of these complex systems, and a large-scale simulation is inevitable. Here, one open issue, related to coherence effects in dynamical processes of complex systems, is still unknown.

Coherence plays an important role in describing correlation properties of quantum matters and understanding quantum phenomena including lasing^9^, Fano shape^10^, superconductivity, superfluidity and Bose-Einstein condensate^11^,^12^, and novel phenomena arising from quantum optics^13^ and attosecond physics^14^. To study these coherence-induced quantum features, dramatic success has also been achieved in preparing and controlling the coherent dominant systems, such as electromagnetically induced transparency in quantum optics^15^ and superfluid-Mott phase transition in condensed matter physics^16^. Recently, with the development of the single coherent attosecond pulse generation, one can coherently control the electron dynamics in the atom on ultrafast timescales. For example, transient changes in the absorption of an isolated attosecond pulse in the presence of a synchronized few-cycle laser pulse were observed in valence electron wavepackets in field-ionized krypton^17^, autoionizing states of argon^18^, and Stark-shifted excited states of helium^19^, where irreversible processes play a tiny role in the ultrafast timescale. More recently, strong field physics of light-matter interactions has expanded from the long- and intermediate-wavelength regime into the X-ray regime, such as the Linac Coherent Light Source (LCLS)^20^ and a series of pioneer experiments and proposals^21^,^22^,^23^,^24^,^25^,^26^,^27^,^28^,^29^,^30^,^31^,^32^,^33^,^34^,^35^,^36^,^37^,^38^,^39^, where one opened a new era of exploring the interaction of high-intensity X rays with complex systems on femtosecond (fs) timescales which are never accessed experimentally before^21^. In a further experiment, a successful coherent free-electron laser radiation pulses in the soft X-ray regime have been generated^40^, which experimentally provides the possibility for investigating the interplay between coherence and dissipation in X-ray-laser-matter systems, such as coherent control of electronic response in the fs core-hole lifetimes^41^,^42^. This system, however, is beyond the semiclassical description of Einstein’s rate equation approach for the traditionally partially coherent free-electron-laser experiments^43^,^44^,^45^,^46^. Then the crucial issue, related to this X-ray laser with an improved temporal coherence, is how to model the ultrafast dynamics of the X-ray-matter systems and understand the underlying physics. To obtain a more precise description of dynamical mechanics, in general, we need a quantum mechanical tool for simulations of its time evolution, such as time-dependent Schrodinger equation. In contrast to the case of low-Z species in dilute gases^47^,^48^,^49^,^50^,^51^,^52^, time dependent Schrodinger equation for complex systems is difficult to tackle directly, due to electron-electron correlations, collision processes, and spontaneous and Auger decay processes. Approximations for the X-ray-laser-matter systems are inevitable, such as master equation approach. In the master equation approach, one can include the important microscopic processes as much as possible and investigate the interplay between coherence and dissipations in the X-ray-matter systems. Instead of a few-level simulations^5^,^6^,^7^,^53^,^54^,^55^,^56^,^57^, one need to simulate dynamics of the complex X-ray-matter systems based on large scale simulations, due to the vast decay channels induced by the intense X-ray laser. As far as we know, this problem is never explored in the framework of quantum mechanics before, coherent dynamics of the complex systems is still unclear, and it is still an open issue whether new phenomena arise from coherence effects in the ultrafast decayed systems.

Here, we establish a general method for describing coherent dynamics of the X-ray-matter systems in the framework of master equation approach by including thousands of states, which was not found in the literature before, and discuss possible experimental implementations such as signatures for coherent evolution of inner-shell electrons. We take dilute atomic gases as examples for discussing coherent dynamics of the rapidly decayed X-ray-matter system. Our studies will provide the basis for understanding the coherence effects in X-ray absorption mechanisms at a fundamental level. In addition to single-photon dominated processes in the present X-ray-laser-matter systems, we study multiphoton dominated processes using this method, and take helium as examples for investigating the transient absorption of an isolated attosecond pulse by helium coupled with a delayed infrared laser pulse.

## Results

### Coherent dynamics of a neon gas irradiated by an X-ray laser

Of our particular interest is to which extent coherence demonstrates a dominant role in the ultrafast decayed nonequilibrium system. Here, we take neon as examples for coherent dynamics of complex atoms induced by an X-ray laser, based on master equation approach by including thousands of atomic levels. The X-ray laser can sequentially excite inner-shell electrons and create a nonequilibrium state decayed through a vast number of channels. To simplify our simulations, we add the atomic orbitals gradually, and mainly focus on dynamics of the lowest-lying orbitals of neon, such as 1 s, 2 s, 2 p, 3 n and 4 n, and inner-shell excited states 1 s^2^n^2^, 1s 2n3p and 1 s2n4p as well, which normally includes atomic levels up to an order of 10^3^ and spans the Hilbert subspace as basis states for dynamics of neon. Here, contributions of both the core-hole Rydberg series and highly excited valence-electron states are neglected, and our selections are supported by the recent R-matrix calculations for inner-shell electrons^58^. After obtaining atomic data, we study the interplay between coherence-induced effects and dissipations in X-ray-atom systems, based on large scale simulations.

**Figure 1:**
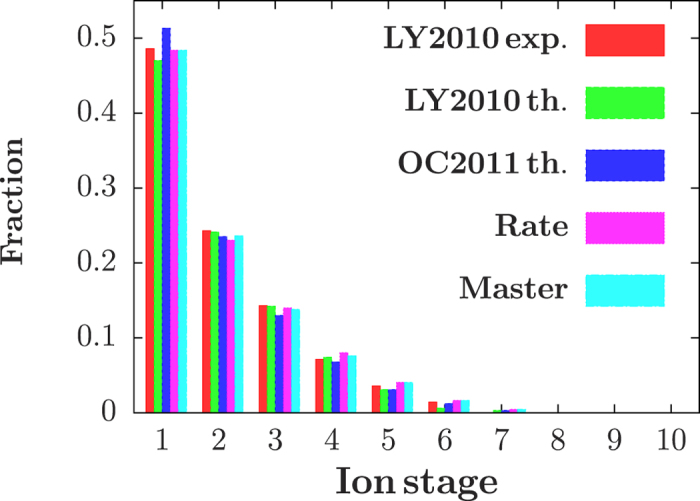
Neon charge-state yields by a far off-resonant beam with photon energy of 800 eV. The calculation is conducted with a Gaussian beam (an intensity of 10^17^ W/cm^2^, 70 fs full width at half maximum). Good agreements between different theories^22^,^43^ and experiments^22^ indicate that coherence, induced by a far off-resonant laser beam, plays a tiny role in the time evolution for the present experimental conditions.

First we review the details of the X-ray free-electron-laser experiment for neon in ref. 23. In this experiment, X-ray pulses with photon energies of 800, 1050 and 2000 eV are injected into a neon gas in the atomic chamber, and they observe rapid photoabsorptions of atomic gases in the fs timescale. As pointed out in the experiment, the dominant processes of electron response are sequential one-photon excitations, ionizations and relaxations of neon triggered by the X-ray beam in the ultra-intense, short-wavelength regime. Here, we take photon energy of 800 eV as examples for investigating the influence of coherence on the sequential multiphoton ionizations of the neon gas, where the photon energy is far below resonant 1s → 3p excitations and it is referred to as off-resonant dynamics of neon. Remarkable agreements are obtained between different theories^23^,^44^ and experiments^23^, as shown in Fig. 1, which indicates that coherence plays a tiny role in the time evolution of neon induced by a far off-resonant X-ray pulse in the present experiments. For comparisons, here we use both Gaussian and flat-topped pulses to simulate the experimentally accessible dynamics of inner-shell electrons of neon, and find that the charge-state distributions are insensitive to the pulse shapes. We remark here that classical rate equation approach was widely used to describe the complex ionization dynamics of atomic systems of neon, krypton and xenon^45^,^59^,^60^,^61^ by including a large number of atomic states. Nevertheless, coherence is totally neglected in these calculations.

**Figure 2:**
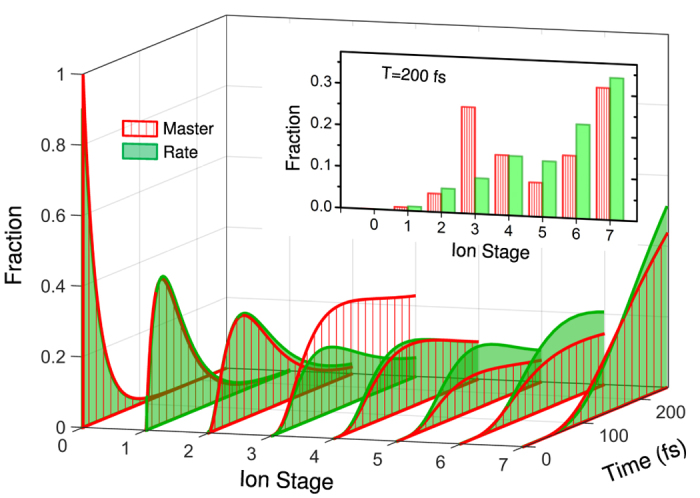
Coherence-induced suppression of multiphoton absorptions: charge-state populations of neon induced by the X-ray laser with an intensity of 2.5 × 10^17^ W/cm^2^ for photon energy at a red detuning of 15 eV with respect to the 1s^2^2s22p^6^ → 1s^1^2s^2^2p^5^3p^1^ transition. Discrepancies between master equation and rate equation are the results of coherence-induced Rabi oscillations and power-broadening effects. Inset: fraction yields for different charge stages of neon obtained by master equation (red) and rate equation (green) for pulse duration of 200 fs.

**Figure 3:**
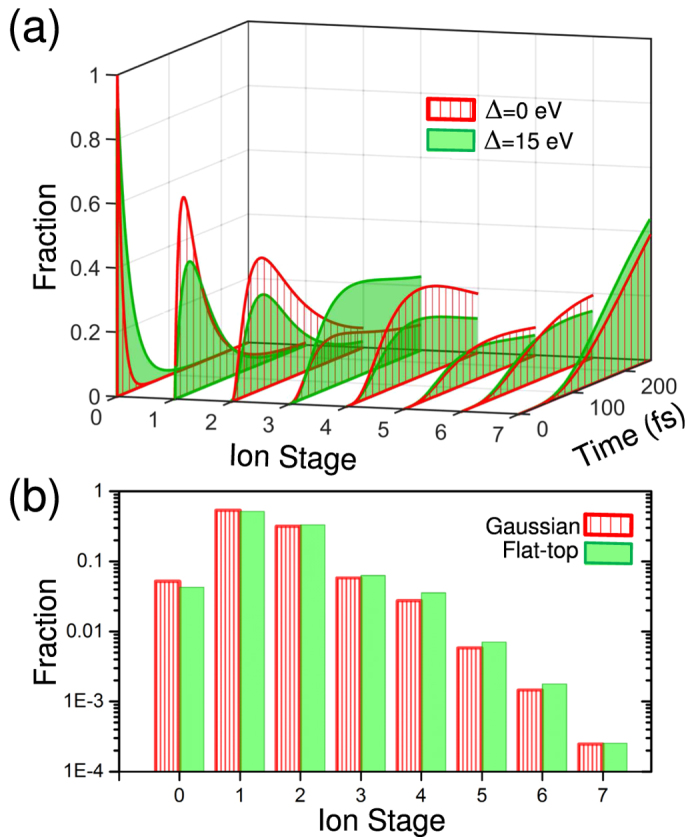
Influence of resonance and pulse shapes on coherent time evolution: (**a**) resonance enhanced ionizations of neon induced by the X-ray laser with an intensity of 2.5 × 10^17^ W/cm^2^, due to 1s → 2p and 1s → 3p transitions; (**b**) pulse-shape independence of charge-state populations of neon induced by a Gaussian pulse of a FWHM duration 25 fs (red) and a flat-topped pulse with the same fluence (green).

For near-resonant photoexcitation, however, coherence plays a pronounced role for the electron dynamics of a neon gas induced by an X-ray laser, such as by recently generated coherent free-electron-laser radiation pulses^40^. Here, we study the time evolution of neon atoms subjected to a strong X-ray laser field with near-resonant photon energies. In Fig. 2, we demonstrate charge-state populations as a function of time for a laser intensity I_0_ = 2.5 × 10^17^ W/cm^2^, which is a typical free-electron laser intensity in the recent experiments, obtained via master equation (Figs 2 and 3) and rate equation approach by using the same energy levels including resonant channels (Fig. 2). The photon energies are chosen near resonant frequency relative to the 1s^2^2s^2^2p^6^ → 1s^1^2s2p^6^3p^1^ transition, such as a red shift of 15 eV (Fig. 2) and the resonant case (Fig. 3), which are both in the photon-energy range of LCLS (800–2000 eV). We find that coherence can suppress the multiphoton ionizations of neon induced by the ultra-intense X-ray pulse. As shown in the inset of Fig. 2, for example, the charge state population of Ne^3+^ after a 200 fs evolution, is twice bigger than that obtained by rate equation approach for incoherent pumping with an intensity of 2.5 × 10^17^ W/cm^2^. The physical origin of the discrepancy in the time evolution is the results of two effects, which are neglected in the Einstein’s rate equation. The first one is the coherence in the inner-shell resonant absorption processes, i.e 1s → 3p excitations for Ne and 1s → 2p for Ne^3+^, Ne^4+^ and Ne^5+^. In contrast to monotonous changes in rate equation approach, real physical processes between different energy levels demonstrate a Rabi-flopping structure, due to the light-atom coupling for the 1 s → 2p and 1 s → 3p transitions. The second one is the power broadening effects, due to the extremely strong X-ray laser field, which can be up to an order of a few eV at a laser intensity of 2.5 × 10^17^ W/cm^2^, based on a two-level estimation 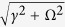 with *γ* and Ω being spontaneous decay rate and Rabi frequency, respectively. Note here that coherence, embedded in the off-diagonal terms of density matrix^53^,^62^, demonstrates an oscillation structure in real-time dynamics, which is normally beyond the time revolution of the X-ray experiments. Instead, one available tool for studying these fast oscillations between different energy levels is fluorescence spectra^7^,^57^,^63^,^64^,^65^ (see appendix). Interestingly, we find that the red-shift case (relative to 1s → 3p transition) ionizes more electrons after 150 fs evolution, since the 1s → 2p resonant transitions enhance ionizations for charge states Ne^3+^, Ne^4+^ and Ne^5+^, whereas the resonant case (with respect to 1s → 3p transition) ionizes electrons fast in the early stage after subjected to the X-ray laser field, since the resonant 1s → 3p excitations dominate photon absorptions of Ne in the first 50 fs, as shown in Fig. 3(a). The influence of temporal pulse shape on the charge-state distributions is also discussed in Fig. 3(b), where comparison has been made between flat-topped (green) and Gaussian pulses (red). We remark here that the temporal pulse shape has limited impact on the charge state distributions, whose conclusion is consistent with those for incoherent pulses^23^.

**Figure 4:**
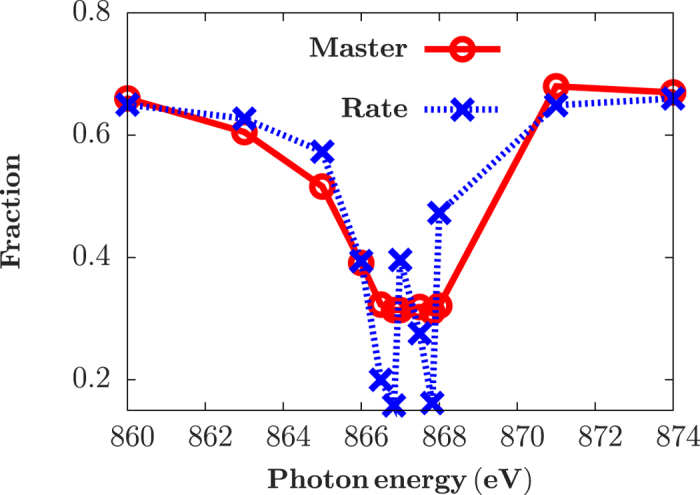
Power broadening effects: Ne fraction in a neon gas subjected to a Gaussian X-ray pulse with an intensity of 2.5 × 10^17^ W/cm^2^ and a FWHM duration of 10 fs, for different photon energies, obtained by master equation (red solid) and rate equation approach (blue dashed).

Next, we address the issue related to power broadening effects in more details. Actually, the broadening effect, resulting in the line shape of a dipole transition in an atom, is a basic feature for describing electron motions and interactions with external fields. The underlying mechanisms of broadening effects are diverse, including natural broadening due to spontaneous decays of excited states, Doppler broadening due to thermal motions of atoms, collisional broadening due to collisions with other atoms or ions, and Stark broadening due to energy shifts induced by an external field. Here, we find that the dominant broadening effect for the dilute neon gas in the LCLS experiment is power broadening (≈1 eV) due to the extremely strong external laser, which is up to 10 times bigger than those from Auger and spontaneous decay processes (≈0.1 eV). Normally the rate equation approach does not include the power broadening effects in the present calculations, while in the master equation approach they are included automatically. In Fig. 4, we show the Ne fractions subjected to a Gaussian X-ray laser with an intensity of 2.5 × 10^17^ W/cm^2^ and a FWHM duration of 10 fs, where two local minima, obtained from rate equation (blue dashed), correspond to resonant excitations for 1s^2^2s^2^2p^6^ → 1s^1^2s^2^2p^6^3p^1^ and 1s^2^2s^2^2p^6^ → 1s^1^2s^2^2p^6^4p^1^ transitions, respectively. However, these oscillating structures for resonant excitations at different photon energies are smoothen in the results obtained from master equation approach (red circle), due to power broadening effects. Note here that the oscillating structures can occur at other photon energies associated with 1s → np (n ≥ 5) transitions, but are beyond the spectrum resolution in the present experiments and an improved sharp-band X-ray laser is needed in the future.

### Attosecond transient absorption of laser-dressed helium

In contrast to the one-photon and Rabi resonance features of x-ray laser driven inner-shell electron dynamics, attosecond transient absorption of infrared laser-dressed helium is a typical system of multiphoton processes and multi-quantum-path interferences involving valence electrons. Motivated by recent experiments^19^, we consider a dilute helium gas that are excited by an isolated attosecond pulse (XUV) with a central frequency *ω*_*XUV*_ = 22.5 eV and a duration of 100 attosecond (as), together with a delayed infrared pulse with a frequency of *ω*_*d*_ = 1.7 eV and a duration of 6 fs. Compared to the infrared pulse, the attosecond pulse is shorter and can be treated as being locked to the infrared field oscillations at a well-defined time. By changing the delay between the attosecond and infrared pulses, the absorption signal provides the possibility for recording the transient information of the absorption or emission processes of laser-dressed helium, even though the time-integrated response of the system complicates the analysis. In this system, the influence of particle collisions can be neglected, since the atomic gas is dilute, while dissipation processes, such as spontaneous decays, are much weaker than coherent processes. It indicates that the light-matter system is a coherent dominant evolution at the fs timescale.

**Figure 5:**
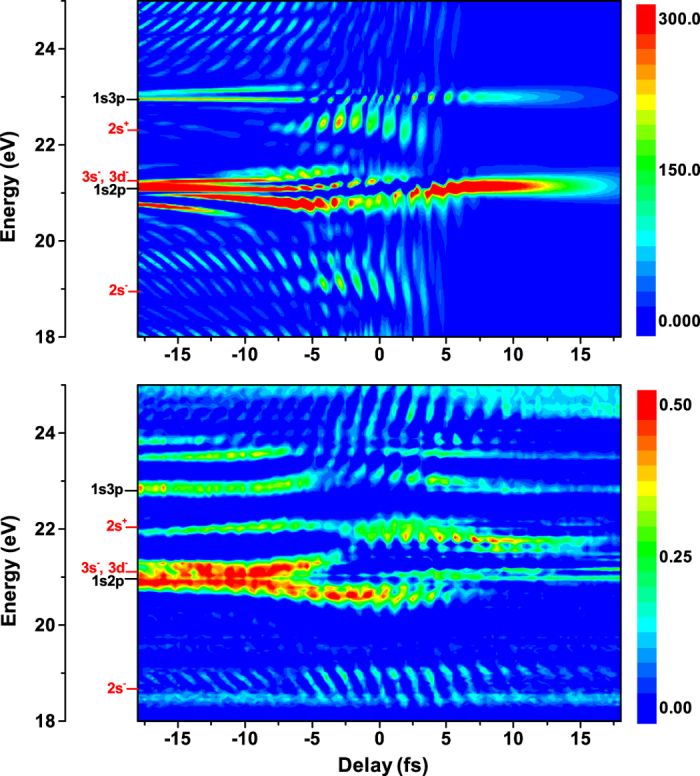
Time-delay-dependent absorbance spectrum of helium with a dressing infrared laser, where comparisons have been made between numerical results (upper) and experimental data^18^ (lower). Here, the time delay is between the dressing laser pulse (*ω*_*d*_ = 1.7 eV, I_d_ = 10^13^ W/cm^2^, FWHM duration of 6 fs) and the attosecond pulse (*ω*_*XUV*_ = 22.5 eV , I_XUV_ = 10^12^ W/cm^2^ and FWHM duration of 100 as).

In our simulations, we focus on the lowest lying excited states of helium, such as 1s2s, 1s2p, 1s3s, 1s3p and 1s3d. The absorption of the attosecond pulse by the system can be described by a frequency-dependent response function^66^,^67^, i.e. 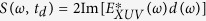 , where *E*_*XUV*_(*ω*) and *d*(*ω*) are the Fourier transforms of the attosecond pulse *E*_*XUV*_(*t*) and the dipole moment *d*(*t*) with delay t_d_, respectively. Here the time evolution of the dipole moment can be obtained from density matrix of the system. In Fig. 5, we show the absorbance spectrum as a function of the time delay for a dressing laser with an intensity of I_d_ = 10^13^ W/cm^2^. Note here that positive delays indicate that the attosecond pulse arrives on the helium gas after the infrared pulse. We find that our results can recover most of the typical features of the transient absorptions of the dressed helium^19^. For example, virtual state absorption features below the ionization threshold [3s^−^ and 3d^−^ (21 eV), and 2s^+^, (22 eV)] and below the 1s2p energy level [2s^+^ (19 eV)] are revealed in the time-dependent simulations. The observed absorption substructures, such as 2s^−^ and 2s^+^, is the results of transient virtual states of the laser-dressed atom, which can be understood in terms of two-color multiphoton absorption processes leading to dipole-forbidden final states after interactions with both XUV and infrared pulses, i.e. absorption of an XUV photon and one or more infrared photons. Therefore, these transient states exist only when both the XUV and infrared lasers interact with the atom. Additionally, we find the absorption signal of each level is modulated on timescales faster than the laser cycle period, such as modulation with a half-cycle periodicity of absorption strength in the vicinity of the 2s^+^, 3s^−^ and 3d^−^, and the physical reason can be explained in terms of the quantum path interference^19^,^66^. In addition to sub-cycle modulations, we find energy level of 1s2p absorption splits dynamically near zero delay, referred to as Autler-Townes splitting, due to resonant coupling between 2p and 3d by the infrared laser pulse.

**Figure 6:**
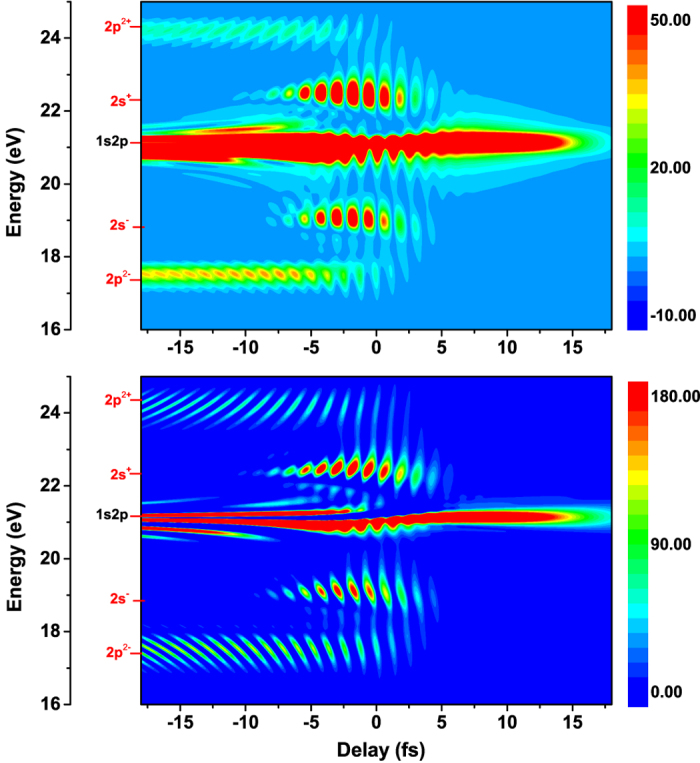
Time-delay-dependent spectrum of helium with a dressing infrared laser based on a three-level model including 1s^2^, 1s2s and 1s2p, where comparisons have been made between polarization (upper) and absorbance spectrum(lower). Here, the parameters are the same as in Fig. 5.

To obtain a clear picture of attosecond transient absorption, we describe the laser-dressed complex system via a three-level model, i.e. the ground state 1s^2^, the first excited 1s2p and the dark state 1s2s. Basically, the ground state 1s^2^ of helium can be excited to the dressed state 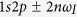 (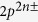) or 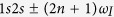 (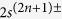) via absorbing an attosecond XUV photon, due to parity conservation. Here, n and *ω*_*I*_ denote the photon number and frequency of the infrared laser, respectively. In Fig. 6, we demonstrate the transient absorption of 2p and 3s, via a three-level model. Here, positive delay corresponds to the attosecond pulse arriving after the center of the IR-dressing field. We observe that the interference fringes appear both at frequency of ±2*ω*_*I*_ distributed symmetrically with respect to the energy 1s2p, and at frequency of ±*ω*_*I*_ around energy level 1s2s. To understand the interference pattern, one has utilize ‘which-way’ (quantum) interference model^19^,^66^,^67^ and optical interference model^68^ to explain the transient absorption oscillations of the low-lying excited states. Here, the inference pattern of 2*s*^±^ arise from the interference between a ‘direct’ pathway 2s^+^(2s^−^), which is populated by the XUV pulse and the dressing field, and an ‘indirect’ pathway, which is populated by the dressed state 2s^−^ (2s^+^) by absorbing two infrared photons. Unfortunately, this model cannot explain the half-cycle oscillations observed in the transient absorption near 17.8 eV, as shown in the lower panel of Fig. 6. By comparing with the delay-dependent polarization spectrum 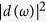 (upper panel of Fig. 6), instead, the interference could result from an optical interference process involving the incident attosecond light and the laser-induced dipole emission^68^, leading to a delay-dependent half-cycle modulation in the measurement.

## Discussion

In conclusion, we establish a general method for describing coherent dynamics of light-matter systems in the framework of master equation approach. This method is a kind of ab initio calculations for the complex systems dressed by an intense laser field, and can resolve real-time dynamics of the far-off-equilibrium systems, based on large-scale simulations. As examples, two typical cases of light-matter interactions, from X-ray-laser interaction to attosecond pulse transient absorption of atomic gases, are studied based on this method. First, we take dilute atomic gases as examples for discussing coherent dynamics of the rapidly decayed X-ray-matter system, based on a thousand state atomic master equation approach, by including coherent pump and incoherent relaxations due to spontaneous and Auger decays. We find that coherence can suppress the sequential single-photon ionizations of a neon gas in the ultra-intense X-ray field, compared to the rate equation approach. The physical reason can attribute to the coherence-induced Rabi oscillations and power broadening effects, which are both neglected in the Einstein’s rate equation. We also find that single-photon ionizations for both outer- and inner-shell electrons dominate the absorptions of a neon gas for the recently typical experiments with a laser intensity of ≈10^18^ W/cm^2^, irrespective of coherence. A typical feature of coherent evolution of inner-shell electrons is Rabi oscillations with a frequency in the order of 10^15^ Hz, which is beyond the current experimental resolutions in the time domain. Instead, we discuss resonance fluorescence spectra for possible experimental implementations for coherent dynamics of inner-shell electrons. Second, we study multiphoton dominated electron dynamics induced by laser pulses. Here, we investigate the transient absorption of an isolated attosecond pulse by helium with a delayed dressing infrared pulse, which demonstrates coherent control over electron dynamics in the multiphoton absorption processes on the sub-laser-cycle timescale.

With the quick development of free-electron lasers, temporal coherence of the X-ray laser is improved experimentally, which provides the possibility for studying time-resolved coherent phenomena in atoms, molecules and solid-state materials^69^. There are still a number of open issues referred to coherent dynamics of complex systems irradiated by an intense X-ray laser. For the methods described here, we are able to discuss hollow-atom signatures and its corresponding coherent dynamics, where the competition between the double- and single-hole generations in coherent evolution is still unclear. Moreover, we can also investigate multiphoton processes of atoms and molecules in gases and solid-dense matters, such as two-photon KK-shell transitions, where the issues are related to non-sequential double ionizations and electron-electron correlations. Finally, in a dense hot plasma environment generated by dense gases or solid-state materials induced by an X-ray laser, the environment is much more complicated, both radiative and particle-collision processes should be taken into account for understanding the phenomena of the system.

## Methods

### Theoretical model

We consider a many-body system, such as dilute atomic and molecular gases^23^,^24^,^29^, and solid-state materials^30^, coupled with incoherent sources such as vacuum, irradiated by a high intensity X-ray laser (without loss of generality, it can also be attosecond and infrared laser pulses). Inner-shell electrons of these atoms and molecules will be excited, forming a far-off-equilibrium system and typically relaxing in a fs timescale via spontaneous photon emission, Coulombic and Auger decay processes. These processes compete with other mechanics, such as processes including coherent photoexcitations and ionizations. Correspondingly, the total Hamiltonian of the X-ray-matter systems can be written as


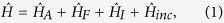


where the total Hamiltonian is the sum of the Hamiltonian 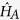 of the many-body system in vacuum, the external field Hamiltonian 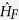 including the laser and chaotic light field, the laser-matter interaction 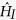, and the chaotic-light-matter interaction 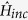.

The Hamiltonian


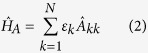


governs the time evolution of the system in vacuum, such as dilute atomic and molecular gases, and condensed materials, with 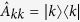 and N being the total energy levels included. Here, 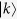 and *ε*_*k*_ denote the eigenstate and eigenvalue of the system, respectively. The Hamiltonian of the external field is given by


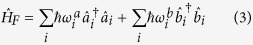


where 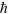 denotes the reduced Planck constant, 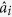 (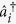) denotes the annihilation (creation) operator that corresponds to the i-th mode of the laser with frequency 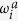, and 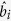 and 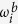 denote those for the chaotic light field.

With the semi-classical treatment for the laser field, the quantization of the light is ignored. Instead the light is considered as a electric field, E(t), which interacts with the i-th dipole d_i_ for the transition between states 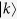 and 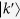 to give


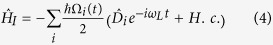


where the transition operator 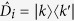, and Rabi frequency 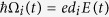 at time t with 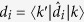. We can also trace out the external field degrees of freedom within the semi-classical treatment indicating the Eq. (3) can be ignored in the simulations. This semiclassical approximation forms the basis of most investigations on the many-body systems both coherently and incoherently coupled by a strong external field.

For magnetic sublevels, we rewrite Eq. (4) in a more explicit form


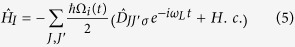


where 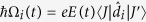 and


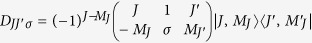
 . Here *σ* denotes the polarization of the external laser, and the dipole operator 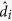 describes a 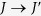 transition between states 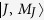 and 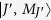 with Zeeman substructures.

The system can also be pumped by an incoherent field, such as the black-body radiation field in a hot plasma environment, and it reads


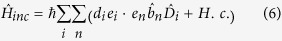


where e_i_ and e_n_ denote the directions of the dipole moment d_i_, the polarization of the incoherent field, respectively. Actually, the contributions of the incoherent field can directly be included in the master equation approach, as discussed in the following.

### Master equation approach

The question is how to theoretically simulate the time evolution of complex systems in the presence of the X-ray laser, described by Eq. (1). In contrast to low-Z atomic and molecular gases^47^,^48^,^49^,^50^,^51^,^52^, the X-ray-matter system is dominated by ultrafast decayed mechanics in the experimental timescales, such as spontaneous decay processes, where one loses the possibility to keep track of the couplings between the system and the environment with infinite degrees of freedom. In solid-state materials, electron-ion, electron-electron and ion-ion collisions occur rapidly and randomly, where screening and broadening effects due to the solid-density environment should be taken into account in dynamical simulations. Hence, a statistical description for the X-ray-matter system is needed, and here we study the time evolution based on a generalized thousand-state master equation approach for the reduced density matrix of the system, where the degrees of freedom of both the environment and X-ray laser have been traced out in a perturbative treatment. The generalized thousand-level master equation for a complex system in Eq. (1), coupled to vacuum modes of the electromagnetic field and irradiated by an X-ray laser and incoherent radiation field, reads


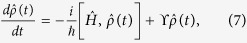


where 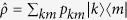 denotes the reduced density matrix operator of the multilevel system, and 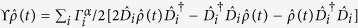 with 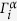 denoting transition rate for the i-th dipole due to quantum process *α*, such as the background radiation pump, spontaneous, Coulombic and Auger decays, photodissociations, and collision processes (see appendix for detailed calculations). To obtain Eq. (7), the standard two-level model coupled with a vacuum environment^53^ has been extended to a multi-level hole-excited system^56^,^57^. Here, the first term in the right side describes the coherent dynamics of the multilevel system coupled with the laser field, and the second term denotes the incoherent processes, such as spontaneous and Auger decays, transitions due to the black-body radiation field and collision processes. The contributions of the black-body radiation pump are only nontrivial in warm and hot matters, and collision processes can be neglected for the dilute atomic and molecular gases since it occurs in a fs timescale being much shorter than the average particle-collision time. Note that the broadening contributions of the incoherent field-system interactions in Eq. (1) and plasma environments are included to the second term in Eq. (7), while the corresponding energy shifts are incorporated in the energy levels of the system in vacuum. Here, the time evolution of Eq. (7) is performed by polynomial expansion of the propagator for the open system in time *t*, and the accuracy of this method is given by truncations of the power series. A parallel version of the code is developed to speed up the numerical simulations. We remark here that the thousand state master equation has unique features, which needs large-scale simulations for propagating in time a matrix of 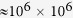 and is not trivial to solve directly, such as stability of numerical linear algebra and parallel procedure demanding.

There are two possible ways to take photoionization processes into account in our simulations. While we treat the ionization as incoherent processes by adding photoionization cross section in the incoherent terms for the low intensity X-ray laser, we consider ionization as coherent processes for multiphoton dominant processes, which feature analogies to bound-state transitions of the system. For multiphoton dominant processes, the ionized state composing of the residual system and ionized electrons reads 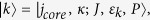 where 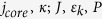 denote the angular momentum of the residual system, relativistic angular momentum of free electrons, total angular momentum, total energy and parity of the system, respectively. In the physical systems, these states can be populated by multiphoton excitations. Considering the large amount of continuous states, selection rules should be used to solely include dominant states, such as degenerate initial, intermediate and finial continuous states connected by multiphoton energies, and at the same time neglect states detuned from resonance excitations since the finite time duration of the X-ray laser implies a finite Fourier width for the bound-free or free-free transitions. These selections are a good approximation for hydrogen in the presence of strong laser beams (see appendix), and therefore we anticipate that the dominant states for ionizations of a complex system should be similar.

The present approach can be applied to many different practical open quantum systems, which are widely treated qualitatively before by a few-level master equation approach. As an example, we apply our method in attosecond physics, and investigate the transient absorption of an isolated attosecond pulse by helium coupled with a delayed infrared laser pulse, even though dissipation processes plays a tiny role in this case and coherent coupling dominates electron dynamics on the sub-laser-cycle timescale. Note that the open quantum system discussed here is based on the Markovian approximation, namely the system time scale is much slower than the one of the environment. When the time scale of the system is comparable to that of the bath, the dynamics of the open system cannot be captured by the approximate memoryless Markovian master equations, but it requires a general non-Markovian description^70^.

Complementary to the multilevel master equation, in this work we employ a degenerate master equation approach to explore the physics of Eq. (1), which is computationally more affordable. Here, the Rabi frequency is 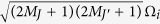 and the decay rate is defined as a total transition probability from one upper state 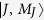 to all the degenerate lower states of the level 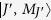.

## Additional Information

**How to cite this article**: Li, Y. *et al.* Coherence and resonance effects in the ultra-intense laser-induced ultrafast response of complex atoms. *Sci. Rep.* **6**, 18529; doi: 10.1038/srep18529 (2016).
